# Reduction of discrepancies between students and instructors in the assessment of practical tasks through structured evaluation sheets and peer feedback

**DOI:** 10.1038/s41598-024-51953-4

**Published:** 2024-01-17

**Authors:** Mozhgan Bizhang, Havre Adib Shaban, Andreas Vahlenkamp, Stefan Zimmer, Andreas Möltner, Jan Ehlers

**Affiliations:** 1 Faculty of Health, Department of Operative Dentistry and Preventive Dentistry, School of Dentistry, Witten/Herdecke University, 58448 Witten, Germany; 2 Faculty of Health, Department of Prosthodontics and Dental Technology, School of Dentistry, Witten/Herdecke University, 58448 Witten, Germany; 3 Center of Excellence for Assessment in Medicine, University of Heidelberg, Grabengasse 1, 69117 Heidelberg, Germany; 4 Chair of Didactics and Educational Research in Health Care, Witten/Herdecke University, 58448 Witten, Germany

**Keywords:** Health occupations, Medical research

## Abstract

The aim of this study was to reduce discrepancies between students and instructors in a preclinical dental course by employing structured peer feedback based on a detailed evaluation sheet. In a crossover study of dental students (n = 32), which compared peer feedback using an evaluation sheet (test) with the traditional method (control), participants completed tasks involving cavity and partial crown preparation. The practical tasks were scored numerically on a scale ranging from one (excellent) to six (failure). The amount of feedback provided by the instructor was also recorded. Statistical analysis was conducted using Wilcoxon signed-rank tests (p < 0.05). Regarding cavity preparation, no statistically significant difference was observed (median (25th–75th percentile)) between the grades received by the test (2.00 (1.50–3.00)) and control groups (2.25 (2.00–3.00)). However, the grades pertaining to partial crown preparation exhibited a statistically significant difference between the test (2.25 (2.00–2.50)) and control (2.50 (2.00–3.00)) groups. LimeSurvey and five-finger feedback were used to assess satisfaction with the new method, revealing that most students found the evaluation sheet and peer feedback to be effective. Within the limitations of this study, structured peer feedback using the evaluation sheet positively impacted grades pertaining to partial crown preparation, requiring less instructor feedback.

## Introduction

A significant component of preclinical dental education involves practical courses. As part of these practical courses, the students acquire the necessary knowledge and skills by practicing on phantom models; this process is intended to enable them to apply these skills while treating patients in their clinical courses. An integral part of the evaluation of the effectiveness of teaching methods in this context is not merely the assessment of student skills by instructors but also self-assessment by the students. Self-assessment is a relevant aspect of dental teaching, as it encourages students to take responsibility for their work and improve their practical skills^1,2^. Previous studies have shown a significant improvement in students' self-assessment skills during their preclinical years^3,4^; on the other hand, other studies have also shown discrepancies between the self-assessment abilities of the students and the lecturer’s assessments^5–7^. These discrepancies between the student’s and lecturer’s evaluations were highlighted in a recent study^8^. Six practical tasks were double-assessed by two instructors and two students using both visual criteria and predefined assessment criteria. The results indicated a high degree of intrapersonal agreement between instructors and students when predefined assessment criteria were used, unlike the case in which the assessment was performed solely through visual inspection^9^. In another study, 55% of the students in their final year of dental school participated in a training course on peer assessment, peer feedback and self-reflection. Compared to the control group (without any intervention), the trained students exhibited a statistically significant increase in their ability to reflect critically. In addition, the use of a structured protocol for peer assessment and feedback resulted in the improvement of students' academic skills^10^. Furthermore, recent research has also emphasized the fact that peer review cannot completely substitute for the instructor’s feedback^11^. A great deal of data has highlighted the importance of using appropriate guidance (evaluation sheets), which should preferably be standardized, to improve self-assessment^12^. A study that aimed to evaluate a newly developed evidence-based model of feedback (MOF) featuring six key steps pertaining to patient safety showed that the introduction of this model resulted in effective feedback even in complex and challenging situations^13^.

Dental schools in Germany feature a 5-year (10-semester) training program. The first five semesters involve general sciences and preclinical training, while the last five semesters focus on medical science and the treatment of patients. Preclinical training is essential for students’ ability to develop the practical skills necessary to patients later in their careers and to judge the quality of their work.

This study aimed to reduce discrepancies in the assessment of practical tasks between students and instructors with the help of structured peer feedback based on a detailed evaluation sheet in the context of a practical preclinical course, in which context the treatment group was compared to the control group, who were assessed using the traditional method. The scores of students in these two groups on practical tasks were compared. The null hypothesis was that the grades and number of feedbacks from the instructor who used structured peer feedback based on an evaluation sheet for the two tasks (i.e., 1a and 1b, pertaining to the preparation of a cavity to receive a composite filling for an anterior tooth and a posterior tooth, respectively, and 2a and 2b, preparation for a partial gold or ceramic crown, respectively) would be equal to those of students in the control groups.

The first hypothesis was that the number of feedbacks obtained by students in the test groups (who used an evaluation sheet focused on structured peer feedback) would be less than those obtained by students in the control groups for two tasks (i.e., 1a and 1b, cavity preparation for a composite filling, and 2a and 2b, partial crown preparation for a molar). The second hypothesis was that the students in the test groups would require higher scores than the students in the control groups.

## Results

### Participants and general data

A total of 32 fifth-semester students (mean age = 24.4, SD = 2.7; 21 females, 11 males) participated in the structured peer assessment and peer feedback protocol.

### Students’ scores on the practical tasks and number of feedbacks from Instructors

The amount of feedback provided by the instructors between the test and control groups exhibited statistically significant differences between the two separate tasks (1. cavity preparation for composite filling and 2. preparation for partial crown). Table 1 presents the mean (SD), median and 25th–75th percentile for the test and control groups between the two tasks, i.e., cavity preparation and partial crown preparation (Wilcoxon signed rank test, p < 0.05).

**Table 1:** Descriptive statistics of the amount of feedback provided by the instructor for cavity preparation and for the preparation of the partial crown between the test and control groups.

	Amount of feedback provided by the instructor; cavity preparation	Amount of feedback provided by the instructor; cavity preparation	Amount of feedback provided by the instructor; partial crown preparation	Amount of feedback provided by the instructor; partial crown preparation
	Test group	Ctrl group	Test group	Ctrl group
Mean ± SD	1.26 (0.51)^a^	1,65 (0.84)^a^	1.66 (0.61)^b^	2.31 (0.93)^b^
Median (IQR)	1.00 (100–1.00)	2.00 (1.00–2.00)	2.00 (1.00–2.00)	2.00 (2.00–3.00)
Wilcoxon p	p = 0.029		p = 0.002	
Effect size	0.39		0.73	

(Mean, (SD = standard deviation), median and IQR (25th–75th percentile), effect size).

^a,b^The same letters in a row indicate a statistically significant difference for each task (^a^cavity and ^b^preparation of partial crown) (Wilcoxon signed rank test, p < 0.05).

The traditional evaluation sheet focuses on two steps of the cavity preparation process: the preparation of the characteristics of the cavity without any detailed information and the maintenance of the integrity of neighboring teeth. The new sheet provides information regarding the characteristics of proximal contact (width and depth), the integrity of neighboring teeth, expansion and cavity design (width and depth), surface smoothing and bevel for anterior and posterior preparation. The traditional sheet for partial crown preparation focuses on the following points: anatomically correct preparation, separation of contact points, the maintenance of the integrity of neighboring teeth, preparation characteristics and surface smoothing. The new evaluation sheet included the following criteria: cavity size (width and depth), cavity wall angulation, cavity floor (characteristics), proximal box depth and wall angle, proximal box floor (axis, surface smoothing), proximal contact (size), adjacent teeth integrity, and occlusal reduction, taking into account occlusal relief.

**Figure 1:**
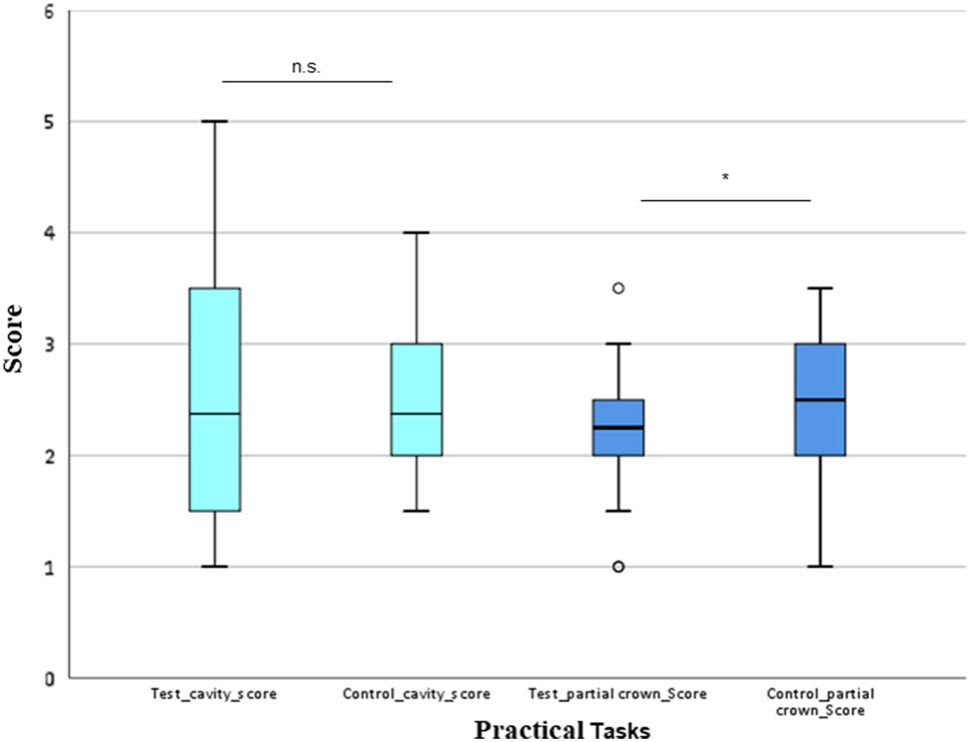
Boxplot of the scores for the cavity preparation (1a, 1b) and partial crown preparation (2a, 2b) tasks across the test and control groups. *P < 0.05: Wilcoxon signed rank tests indicate statistically significant differences, outliers.

The scores for cavity preparation for composite filling (task 1) and preparation for the partial crown (task 2) are shown in Table 2 and Fig. 1. The scores exhibited no statistically significant differences between the test and control groups with regard to cavity preparation for composite fillings but did exhibit statistically significant differences for gold and ceramic partial crown preparation (Wilcoxon signed rank test, p < 0.05).

**Table 2:** Descriptive statistics of the scores for cavity preparation and partial crown preparation across the test and control groups.

	Cavity test	Cavity ctrl	Partial crown test	Partial crown ctrl
Mean (SD)	2.35 (1.05)	2.44 (0.62)	2.20 (0.54)^a^	2.49 (0.72)^a^
Median (IQR)	2.00 (1.50–3.00)	2.25 (2.00–3.00)	2.25 (2.00–2.50)	2.50 (2.00–3.00)
Wilcoxon p	p = 0.409		p = 0.041	
Effect size	0.09		0.42	

(Mean (SD = standard deviation), median and IQR (25th–75th percentile), effect size).

^a^The same letters in a row indicate a statistically significant difference with regard to the partial crown task (Wilcoxon signed rank test, p < 0.05).

### Five-finger feedback

The students’ feedback showed that most of them were satisfied with the new method (peer feedback using the evaluation sheet). In addition, they indicated that they wished that this method had been used earlier in the year, i.e., from the beginning of the course. On the other hand, they indicated that the new method was very time consuming, that the evaluation sheet should be shortened, and that additional space for peer comments should be provided. Furthermore, with regard to time conservation, they felt that the administrative tasks (the information event and the distribution of peers) should have been addressed before the practical course began. Table 3 shows the descriptive statistics regarding students’ answers to the questions of “what went well”, “what we would like to keep”, and “what did not receive enough attention” in the five-finger feedback regarding cavity preparation and partial crown preparation. Their responses to the question of "what could be improved" were similar to their responses to the question of "what we would like to keep". Similarly, their responses to the question of "what went wrong" mirrored their responses to the question of "what did not receive enough attention". Since students did not provide distinct answers to these questions, the presentation omits the responses to these two questions due to the identical results obtained. The results of the five-finger feedback indicated that the majority of students rated the evaluation sheet and peer feedback as effective with regard to both partial crown preparation and cavity preparation, accordingly expressing the desire to continue using this method. However, they expressed preferences for fewer intermediate stages and more time.

**Table 3:** Descriptive statistics regarding responses to the questions of “what went well”, “what we would like to keep”, and “what did not receive enough attention” with regard to cavity and partial crown preparation (number and frequency of answers).

Answer n (%)	Evaluation sheet	Peer feedback	Peer feedback and evaluation sheet	No answer
Question: What went well				
Cavity	9 (29%)	12 (38.7%)	9 (29)	1 (3.2%)
Partial crown	7 (22.6%)	8 (25.8%)	13 (41.9%)	3 (9.7%)

Answer n (%)	Evaluation sheet for self-regulation	Peer feedback makes sense	Evaluation sheet for self-regulation und peer feedback makes sense	No comment
Question: What we would like to keep				
Cavity	17 (54.8%)	7 (22.6%)	4 (12.9%)	3 (9.7%)
Partial crown	13 (41.9%)	7 (22.6%)	6 (19.4%)	5 (16.1%)

Answer n (%)	Many items	Time	More practical tasks	No comment
Question: What did not receive enough attention				
Cavity	1 (3.2%)	2 (6.5%)		28 (90.3%)
Partial crown	0 (0%)	2 (6.5%)	2 (6.4%)	27 (87.1%)

### Students' satisfaction

Twenty-two of the 32 students completed the online survey after the practical course. The results are presented in Tables 4 and 5. Most students reported that they liked the new method. However, they found that the time available for the new method was short and that the evaluation sheet was too long. They were able to implement the method well, and it was enormously helpful with regard to understanding the workflow. They found that this method was more versatile than the traditional method. The students, however, were not happy with the time management associated with the new method. Seventy percent of the students indicated that they would like to involve additional new teaching methods in dental education, whereas 30% preferred traditional lectures. The results of the LimeSurvey showed that the mean Likert scale scores for all feedback questions, with one exception, ranged from 1.26 to 2.65 (1 = strongly agree and 2 = agree), and all responses were below a score of 3 on the Likert scale (undecided). Students expressed agreement with the new procedure and recognized its advantages. One question attained a mean score of 3.13: "After the feedback, I have achieved better time management to carry out the task". The notion of time constraints was noted in both the LimeSurvey and the five-finger feedback.

**Table 4:** Means (standard deviation, SD) of the analysis of the LimeSurvey questions regarding students’ satisfaction (ranging from strongly agree = 1 to strongly disagree = 5).

	Question	Mean (SD)
1	I completed the task myself under the supervision of the instructors	1.39 (0.99)
2	I can now perform the task independently without instructors	1.64 (1.03)
3	The student gave meaningful feedback that I can use in my current and future activities	1.78 (0.7)
4	I was satisfied with the clarity of the feedback and the explanations	1.93 (0.87)
5	I was satisfied with the atmosphere and environment for giving feedback	1.76 (0.97)
6	I was satisfied with the extent and quality of the instructors' feedback on my performance	1.78 (1.00)
7	I received constructive feedback from the students on the task that I completed	1.48 (0.9)
8	At the beginning of the semester, my practical skills in the subject were at a high level	2.22 (1.00)
9	By the end of the semester, my practical skills in the subject were at a high level	1.91 (0.95)
10	I integrated my own learning steps through feedback and organized my learning process myself	1.78 (0.8)
11	The instructors' feedback and suggestions for improvement were helpful	1.43 (0.73)
12	I felt that the instructors took my special skills and difficulties into account	2.17 (1.37)
13	The course encouraged collaboration and mutual support among students	1.43 (0.73)
14	I am satisfied with the feedback provided and the results of the feedback	1.52 (0.59)
15	I find it easier to perform the practical task now than I did at the beginning of the course	2.39 (1.23)
16	Through the feedback, the students motivated me to improve my understanding of the work process	2.39 (1.08)
17	After receiving feedback, I achieved better time management with regard to completing the task	3.13 (1.22)
18	The process strengthened my self-critical view, enabling me to evaluate my own task	1.91 (1.08)
19	The instructors took my opinion into consideration when providing feedback	1.78 (1.04)
20	With the help of the feedback, I was able to acquire further knowledge of the subject	2.09 (0.85)
21	My knowledge of the subject improved after the course	2.13 (1.01)
22	Perception of the atmosphere: the feedback increased my motivation and willingness to learn	2.04 (1.07)
23	My personal motivation was increased by the feedback	2.22 (1.28)
24	I gained more self-confidence due to the course	2.65 (1.4)
25	For me, the benefit of what I learned was sufficiently great to make it worth the effort	2.13 (1.1)
26	I rarely became bored in class. I was satisfied with the working atmosphere in the course	2.26 (1.42)
27	I have the feeling that my task was judged more objectively than last semester	1.26 (0.54)
28	Which method would you like to use in the future? 1 = structured peer feedback using the evaluation form or 2 = the old form without peer feedback	1 (70%) 2 (30%)

**Table 5:** Analysis of the students’ satisfaction according to the LimeSurvey (free text).

Please indicate your suggestions for improvement; Answer (mentioned one or several times)
More time, more preparation using the evaluation sheet, suitable for earlier semesters, at the beginning of the topic “Preparation”, the evaluation sheet should be shortened
Please indicate the positive aspects of the new method; Answer (mentioned one or several times)
Peer feedback, improved relationships with other students, peer feedback using the evaluation sheet, more peer feedback and less instructor feedback than the traditional method, the waiting time for the instructor was shorter, self-regulation, more details promoted a better understanding, evaluation sheet
Please indicate the negative aspects of the new method; Answer (mentioned one or several times)
Further details for preparation rules should be provided, more time, differences in constructive feedback among peers

The instructors answered the questions included in the LimeSurvey. They reported an increase in the responsibility and self-regulation of students as well as an increase in the efficiency of the course, which enabled it to obtain better results. Instructors found the evaluation sheet to be suitable for the students and for their own calibration. The sheet was invaluable with regard to expressing teaching opinions clearly and simply to the students. The negative aspects included time pressure, inequality within the groups and the increased time requirements associated with the implementation of this method. The instructors suggested that the evaluation sheet should be supplemented with a graphical representation, that the text should be shortened and that more time should be made available for the implementation process. The instructors also preferred the new method of education.

## Discussion

This study evaluated the effectiveness of structured peer feedback using an evaluation sheet in the context of a preclinical course for third-year undergraduate dental students who were required to perform practical tasks. The practical tasks thus performed and evaluated focused on preparation for direct and indirect restorations. Dentistry in Germany is currently a female-dominated profession, with the female-to-male ratio being approximately 2:1. Hence, 21 women and 11 men participated in this study. The structured peer feedback using an evaluation sheet indicated an increase in scores for the preparation of a partial crown but not for scores for cavity preparation for a composite filling. The 3rd-year students found the preparation for a direct restoration to be easy despite the fact that the lecture describing the topic had taken place in their 2nd year of dental school. However, the preparation for a partial crown is more complex, and peer feedback using the evaluation sheet was found to support the students more effectively than the traditional method. Accordingly, we are inclined to believe that the peer feedback and evaluation sheet for composite should be used for 2nd-year students. One study reported that students consistently benefited from learning the criteria associated with preparation for a gold restoration, followed by composite and ceramic preparation. Learning these criteria was shown to be beneficial with regard to feedback, and the instructors rated the criteria as helpful for task evaluation and feedback efficiency^9^. Similarly, the instructors who participated in our study found the evaluation sheet to be extremely valuable. Thus, they expressed the desire to apply the evaluation sheet to the calibration of the instructors as well. Furthermore, the evaluation sheet plays an important role in teaching the students to be better self-evaluators. When performing the first task (cavity preparation), the students encountered problems with regard to distributing the evaluation sheets and establishing pairs of peers. Both of these duties were new to the students in the practical course; therefore, they required more time to organize themselves. On this basis, unequal conditions between the 1st and 2nd tasks can possibly be noted in a certain respect. One study reported good results with regard to self-assessment in the context of a clinical dentistry practical training course on communication skills for fourth-year dental students^14^. The students in the test group identified self-evaluation as an advantage in dental teaching that can support effective self-directed learning. Other studies have shown that the best results in terms of high reliability were obtained when the examiner’s evaluation sheet was used^15,16^. It is important to ensure that the conditions under which the investigation takes place, such as time, instructors, identical tasks and phantom rooms, should be the same between the test and control groups. A crossover design was chosen to prevent bias, i.e., in this case, the scores on the two separate tasks, as evaluated by two instructors. Thus, continuous reflection on one’s own task and hence on one’s own abilities was promoted. The literature has shown that peer feedback in an institutional environment supports success during the stressful period of medical training and residency^17–19^. Simultaneously, it fosters professional behaviors, especially at the interpersonal level.

The student evaluations revealed that most of the students who participated in our study criticized the lack of transparency or objective assessments from instructors in the context of preclinical practical dental courses. To develop clinical competence with regard to the treatment of patients in clinical courses, the students required self-assessment skills and the ability to provide constructive feedback^20,21^. The amount of feedback provided by the instructors was significantly lower in the test group than in the control group for both practical tasks. A review of three meta-analyses regarding the self-assessment of performance by medical students reported a moderate correlation between self-assessment and criterion scores. The students were able to self-assess their performance only moderately, although their accuracy improved in later stages of medical school^22^. Our study, with the help of the main results revealed through the comments on the five-finger feedback and the LimeSurvey, showed that the students were satisfied with the evaluation sheet and peer feedback and that they developed a better sense of self-evaluation. We hypothesized that the new method led to greater agreement between the requirements of the instructor and those of the students. Score for the partial crown preparation were significantly higher for the test group than for the control group. Timing and detailed feedback play major roles in learning success^23^. The students who participated in this study received immediate, detailed, clear, personalized, effective feedback. Peer assessment gives students the opportunity to view their peers from a different perspective and to develop an effective social collaborative learning community that inspires critical thinking through problem solving^24^. A major problem reported by the students pertained to time management. In this study, we initially stipulated a three-hour time limit. Based on student feedback, no such time limit will be mandated in future iterations. Since this procedure was applied for the first time, more organizational issues were encountered in the first implementation. The second implementation, however, was better. We will work on improving the organization of this procedure for future implementations. Another point mentioned by the students appertained to their desire for further independence while performing the practical tasks. This approach will also be used in the assessment by instructors to ensure that the evaluation is more objective. Student satisfaction will be assessed in the next iteration to validate the present results, and if necessary, corresponding tasks will be reduced. The students’ scores on the satisfaction scale were below 3, indicating that they were satisfied with the new procedure.

Students’ suggestions for improvement of the new method, such as “the new method is more suitable for previous semesters” and “more time-consuming”, will be considered for the development of new teaching methods pertaining to other practical tasks and for dental students from the very beginning of their first semester. The feedback provided by the students and instructors played a major role in our study, as our aim is to ensure that this form of teaching is implemented in practical courses, primarily in preclinical contexts and thereafter in clinical teaching at our university.

### Limitations of this study

As two instructors performed this study, existing differences in teaching experience can be assumed. Furthermore, the tasks were not scored anonymously by these two instructors, which could easily have biased the results. On the other hand, both instructors had more than three years of work experience pertaining to this preclinical course, and the crossover design was selected to reduce bias. The second limitation was related to the small number of students who participated in the study.

## Methods

### Ethical clearance

The local ethics committee of the University of Witten/Herdecke granted ethical approval for the study (number: S-94/2022). The study was performed in accordance with the current version of the Declaration of Helsinki as well as relevant regulations governing human participants and data protection laws. All participants provided written informed consent. The data used in this study were collected from early November 2022 to January 2023.

### Study design

The study employed a crossover design, including two tasks that were performed in two separate rooms. Thirty-two students in their fifth semester (3rd year, preclinics) participated in this study and were supervised by two well-trained dental instructors. These fifth semester students must take the second state examination at the end of the semester and must be well prepared to assess their work independently and adequately. Preclinical practical courses are mandatory courses that serve to teach students all the skills required for the independent treatment of patients in the clinical course. This study evaluated the students as they performed two practical tasks, i.e., 1a and 1b, which featured a similar level of difficulty (direct composite filling restorations (front vs. posterior tooth)) and 2 and 2b which focused on indirect restorations (gold and ceramic restorations on molars). The cavities in the anterior and posterior regions are not 100% identical. Generally, treating posterior teeth is more challenging than treating anterior teeth. Similar levels of difficulty were present in both groups. We found the workload to be comparable; while localization may be thought to be more challenging in the posterior area, achieving an optimal cavity with two bevels for better light reflection in the anterior area, in order to meet high aesthetic standards, is equally demanding. The use of bevels in anterior composite preparations increases retention and improves esthetics (tooth shade match, blending effect). Therefore, these two types of cavities were selected in this study. The cavities of the direct composite restorations had two surfaces: mesio-labial in the front teeth and mesio-occlusal in the first molar. The cavities pertaining to the indirect partial crown restorations had five surfaces: mesial, distal, occlusal, buccal and lingual. Students in both groups were given three hours to complete each task. Each student was included in the control group and the test group in turns. The distribution of peer pairs was performed randomly. The random allocation of a peer was based on two draws from the numbers 1 to 16. Each student drew a number, and students with the same number were assigned as peer pairs. The instructors used the old evaluation sheets for the test and control groups. The students in the test groups used structured peer feedback with an evaluation sheet.

#### Task 1: Cavities for composite fillings

1a: Sixteen students from Room 1 worked on composite fillings for cavities in the posterior area using the new method of "peer feedback using a standardized evaluation form". The 16 students from Room 2 performed the same cavity preparations for composite fillings in the posterior area using the traditional method "without peer feedback or the standardized evaluation form".

1b: Sixteen students from Room 1 worked on composite fillings for cavities in the anterior area using the traditional method "without peer feedback or the standardized evaluation form". The 16 students from Room 2, on the other hand, utilized the new method of "peer feedback using a standardized evaluation form" for cavity preparations in the anterior area.

#### Task 2: Preparations for partial crowns

2a: Sixteen students from Room 1 performed preparations for a gold partial crown using the new method of "peer feedback using a standardized evaluation form”. Meanwhile, the 16 students from Room 2 performed the same preparations for a gold partial crown using the traditional method “without peer feedback or the standardized evaluation form".

**Figure 2:**
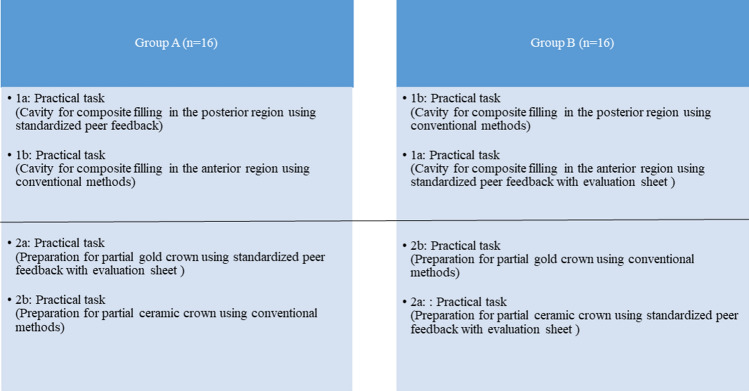
Crossover design.

2b: Sixteen students from Room 1 prepared for a ceramic partial crown using the traditional method "without peer feedback or the standardized evaluation form". On the other hand, the 16 students from Room 2 performed identical preparations for a gold partial crown using the new method of "peer feedback using a standardized evaluation form" (Fig. 2).

Prior to the commencement of the study, all the students and instructors received detailed information regarding the project (a process which took approx. 45 min). The students rated their tasks using both nonstandardized feedback (“traditional method”) and standardized peer feedback based on evaluation sheets (“new method”). The traditional method required the instructor to provide students with one-to-one descriptive feedback and specific advice regarding the quality of their practical task performance and steps they could take to perform better. The students were not provided with any written criteria for each workflow. The criteria were described by the instructor during a lecture. The traditional concept of evaluation was used for the control group. In conformity with the crossover design of the study, each student shifted between the test group and the control group twice. The practical tasks were scored on a scale ranging from one to six; a score of “1” was assigned to the best practical work, and a score of “6” indicated failure. For this study, a score of “5” was also considered to indicate failure. Students in the test group used the evaluation sheets to assess their task, and simultaneously, peers provided feedback to their fellow students using the evaluation sheet. The peers did not assign any grades to their fellow students. The evaluation sheet was very detailed, and all steps were described extensively, e.g., the width and depth of the relevant cavity and partial crown preparations. The students in the control group were instructed in the guidelines for cavity and partial crown preparations in the corresponding lectures. They did not use evaluation sheets for guidance. The students in the control group were taught using the traditional method. In accordance, they received subjective feedback from their instructors. This feedback was, however, not always consistent across instructors. The instructors used a score sheet containing three items: preparation characteristics, integrity of the adjacent teeth and an anatomically correct preparation. All these items lacked details or descriptions. This form of evaluation by instructors is traditional. Therefore, the students did not find these evaluations to be objective. At the conclusion of the task, the instructors assigned a school grade. A grade of “6” indicated grave preparation errors, e.g., if the phantom models or neighboring teeth were damaged. A grade of “5” indicated inadequate completion of the task.

The instructors evaluated the students´ practical tasks using their own evaluation sheets. They assessed the composite fillings for cavities (1a, 1b anterior and posterior teeth) by inspecting the preparation characteristics and the integrity of the adjacent teeth. The preparation for the partial crowns (2a, 2b) was evaluated by checking for anatomically correct preparation, separation of the proximal contacts, integrity of the adjacent teeth and preparation characteristics. All the criteria lacked detailed descriptions. Another person (MB) entered, collected and analyzed the data.

### Evaluation sheet

The new evaluation sheet included six items pertaining to cavity preparation (tasks 1a and 1b) and nine items pertaining to the preparation of a partial crown (tasks 2a and 2b). The items described for cavity preparation included (a) proximal contact, (b) distance to neighboring teeth, (c) width and (d) depth of cavity preparation, (e) smoothness of the cavity surface and (f) bevel preparation at the cavity margins.

The following items pertained to the partial crown (gold): 1: separation of proximal contact, 2: proximal box (parapulpal walls, distance to neighboring teeth) 3: no damage to neighboring teeth, 4: occlusal box (depth, width, taper), 5: functional cusp bevel, 6: load-bearing cusp reduction, 7: nonfunctional cusp preparation, 8: preparation of chamfer margins, and 9: smoothness of preparation.

The following items pertained to the partial crown (ceramic): 1: separation of proximal contact (distance to neighboring teeth) 2: no damage to neighboring teeth, 3: occlusal reduction (1.5 mm in the region of the fissure), 4: occlusal isthmus (2.5 mm), 5: rounded transitions from the occlusal cavity floor to the parapulpal walls, 6: cavity angle 6°–10°, 7: surface angle to the tooth surface approximately 90°, 8: rounded preparation, and 9: smoothness of preparation.

Every item included three subunit descriptors: optimal and correct, acceptable with minor mistakes and not acceptable with major mistakes. The students and peers assessed the tasks using the evaluation sheet. Thereafter, the instructors scored the tasks. The instructors reviewed and agreed with the evaluation sheet before the study started; however, they did not use it to evaluate the tasks.

(The evaluation sheets can be found in the Supplementary Files S1–S5).

### The five-finger feedback method

The five-finger feedback method was used to provide structured feedback to the students. The aim of the five-finger feedback was to enable students to formulate and solve the various questions that occurred to them during the process of implementing the new method. One of the main advantages of this method pertained to the ability of the students to communicate and interact with the instructors. Every finger is associated with a type of feedback. To enhance the approach, the students were required to use a drawing of a hand, in which context each finger represents one form of feedback used in this method.

- Thumb: what went well.
- Index finger: what could be improved.
- Middle finger: what went wrong.
- Ring finger: what we would like to keep.
- Little finger: what did not receive enough attention.

### Satisfaction questionnaire and self-assessment

The students' satisfaction and self-assessment of their abilities were determined immediately after the practical session using the online questionnaire tool LimeSurvey^25^. For this purpose, the questions proposed Schüttpelz-Brauns et al.^26^ by were modified. The LimeSurvey contained a questionnaire featuring 31 items that students were asked to complete immediately, anonymously and easily using a smartphone. Data were evaluated at the end of the study period. The problems thus revealed and their possible solutions were documented to promote the successful establishment of the structured peer-feedback method over the long term in dental courses. Before starting the survey, students completed an informed consent form to acknowledge the information they had received regarding the survey and their voluntary participation in this research. The survey consisted of demographic questions (sex and age), thirty items that were scored on a 5-point Likert scale (ranging from strongly agree = 1 to strongly disagree = 5), and three open-ended questions pertaining to the students´ perception of the benefits and disadvantages of the peer feedback method. The questionnaire focused on the structure of the course, the materials supplied, and the students’ interest in and satisfaction with the new method.

Data were analyzed after the conclusion of the study. The hypothesis concerning the positive acceptance of the feedback and the application of the evaluation sheets was verified with the help of the LimeSurvey questionnaire. A concluding analysis identified the relevant parameters that may have led to the successful implementation of the "evaluation sheets" in the practical dental teaching course and ways in which this approach can be put to use in the long term. A further intention of this analysis was to establish a foundation for a joint discussion among all relevant parties with the goal of providing recommendations for preclinical dental teaching.

### Data analysis

The sample size was estimated using the program G*Power 3.1.9.2^27^. It was necessary to conduct this study based on a Cohen’s d z ≥ 0.8 (effect size (pre-posttest)^28^ 2.89) and a probability (power) of 80%; the 1-sided t test Wilcoxon signed-rank test (matched pairs) at the 0.05% level was used with a sample size of 13 students per group (based on the minimum asymptotic relative efficiency (ARE) parent distribution). However, to compensate for the dropout rate (individual absences due to illness), the researchers planned to recruit 16 subjects per group.

The data were checked for a normal distribution using Kolmogorov‒Smirnov and Shapiro‒Wilk tests. The Wilcoxon signed rank test was then used to analyze the data collected from the test and control groups. The level of significance was defined as p < 0.05.

## Conclusion

Participants’ first experiences with structured peer feedback using the evaluation sheet can be judged positively in terms of the accuracy of self-assessment with regard to the preparation of partial crowns in the context of a preclinical course for dental students. Within the limitations of the study, the results showed that the use of peer feedback based on a structured evaluation sheet reduced the discrepancies between the evaluations made by the students and those made by instructors. Students who performed partial crown preparations received better scores and less instructor feedback. Therefore, it may be possible to conclude that students can self-reflect on and appraise their tasks more effectively with the help of the evaluation sheet and peer feedback. Self-assessment ability is important because students must learn to evaluate their preclinical and clinical tasks accurately. Further studies featuring more participants are needed to substantiate this hypothesis. The participants in this study were satisfied; however, they would prefer using the evaluation sheet for cavity preparation in earlier semesters.

### Supplementary Information

Supplementary Information 1.

Supplementary Information 2.

Supplementary Information 3.

Supplementary Information 4.

Supplementary Information 5.

Supplementary Information 6.

## Data Availability

The datasets are available in the manuscript and in the supplementary materials in the form of Excel data.
