# Supplementary material for: Reduction of discrepancies between students and instructors in the assessment of practical tasks through structured evaluation sheets and peer feedback
Source: Sci Rep. 2024 Jan 17;14:1514. doi: 10.1038/s41598-024-51953-4 (PMC10794213; doi:10.1038/s41598-024-51953-4)
Supplement: Supplementary file 2 — Supplementary Information 2. [file 41598_2024_51953_MOESM2_ESM.pdf]

**Semester**	**Name of student:**	Stud.	peer
**Task**	**Preparation for a composite restoration - anterior tooth**		
**Cavity**	36 mesial-occlusal		
Proximal contact	1. All proximal contacts are adequately broken (tip of probe is able to be passed through).		
	2. Proximal contacts are broken too broadly (over-extended)		
	3. Proximal contacts are not sufficiently broken (tip of probe cannot be passed through/ under-extended)		
	a) No correction is necessary.		
	b) Correction attempt after feedback was successful.		
	c) Correction attempt after feedback was unsuccessful.		
	d) Feedback was helpful for the correction.		
	e) Correction is no longer possible.		
	f) Feedback was helpful for the analysis.		
Integrity of adjacent teeth	1. No iatrogenic damage to the adjacent tooth/ teeth		
	2. Minor iatrogenic damage to the adjacent tooth/ teeth		
	3. Significant iatrogenic damage to the adjacent tooth/ teeth		
	a) No correction is necessary.		
	b) Correction attempt after feedback was successful.		
	c) Correction attempt after feedback was unsuccessful.		
	d) Feedback was helpful for the correction.		
	e) Correction is no longer possible.		
	f) Feedback was helpful for the analysis.		
Extention and design of the cavity	1. Adequate extent and design of the cavity		
	2. oversized extent design of the cavity		
	3. undersized extent and design of the cavity		
	a) No correction is necessary.		
	b) Correction attempt after feedback was successful.		
	c) Correction attempt after feedback was unsuccessful.		
	d) Feedback was helpful for the correction.		
	e) Correction is no longer possible.		
	f) Feedback was helpful for the analysis.		
Surface smoothening	1. All surfaces are optimally smoothened		
	2. Small areas still need to be smoothened		
	3. (Almost) the entire cavity still needs to be smoothened		
	a) No correction is necessary.		
	b) Correction attempt after feedback was successful.		
	c) Correction attempt after feedback was unsuccessful.		
	d) Feedback was helpful for the correction.		
	e) Correction is no longer possible.		
	f) Feedback was helpful for the analysis.		
Beveling	1. Adequate bevelling of cavity in accordance with the preparation rules		
	2. Bevel is uneven		
	3. Bevel is insufficiently pronounced		
	a) No correction is necessary.		
	b) Correction attempt after feedback was successful.		
	c) Correction attempt after feedback was unsuccessful.		
	d) Feedback was helpful for the correction.		
	e) Correction is no longer possible.		
	f) Feedback was helpful for the analysis.		
