# Supplementary material for: Reduction of discrepancies between students and instructors in the assessment of practical tasks through structured evaluation sheets and peer feedback
Source: Sci Rep. 2024 Jan 17;14:1514. doi: 10.1038/s41598-024-51953-4 (PMC10794213; doi:10.1038/s41598-024-51953-4)
Supplement: Supplementary file 4 — Supplementary Information 4. [file 41598_2024_51953_MOESM4_ESM.pdf]

**Semester**	**Name of student:**	Stud.	peer
**Task**	**Partial crown - gold**		
**Tooth**	46		
**Cavity design**			
Occlusal cavity	1. Adequate extention (1.5-2.5mm depth, 2-3mm width, along the main fissure, slightly conical)		
	2. Over-extention of the cavity		
	3. Under-extention of the cavity		
	a) No correction is necessary.		
	b) Correction attempt after feedback was successful.		
	c) Correction attempt after feedback was unsuccessful.		
	d) Feedback was helpful for the correction.		
	e) Correction is no longer possible.		
	f) Feedback was helpful for the analysis.		
Proximal boxes	1. Adequate extention of the proximal boxes (2mm deep, 3-6° conicity, slightly wing-shaped path)		
	2. Over-extention of the proximal box		
	3. Under-extention of the proximal box		
	a) No correction is necessary.		
	b) Correction attempt after feedback was successful.		
	c) Correction attempt after feedback was unsuccessful.		
	d) Feedback was helpful for the correction.		
	e) Correction is no longer possible.		
	f) Feedback was helpful for the analysis.		
Shoulder on the functional cusp	1. Adequate extention of the shoulder on the functional cusp (1mm)		
	2. Over- extention of the shoulder on the functional cusp (>1mm)		
	3. Under- extention of the shoulder on the functional cusp (<1mm)		
	a) No correction is necessary.		
	b) Correction attempt after feedback was successful.		
	c) Correction attempt after feedback was unsuccessful.		
	d) Feedback was helpful for the correction.		
	e) Correction is no longer possible.		
	f) Feedback was helpful for the analysis.		
Reduction of the non-functional cusp	1. Adequate reduction the non-functional cusp (inner and outer surface)		
	2. Over- reduction of the non-functional cusp		
	3. Under- reduction the non-functional cusp		
	a) No correction is necessary.		
	b) Correction attempt after feedback was successful.		
	c) Correction attempt after feedback was unsuccessful.		
	d) Feedback was helpful for the correction.		
	e) Correction is no longer possible.		
	f) Feedback was helpful for the analysis.		
Cavity taper according to the direction of insertion	1. Adequate divergence corresponding to athe direction of insertion (3-6°)		
	2. Over-divergence (>6°)		
	3. Inadequate divergence (<3°)		
	a) No correction is necessary.		
	b) Correction attempt after feedback was successful.		
	c) Correction attempt after feedback was unsuccessful.		
	d) Feedback was helpful for the correction.		
	e) Correction is no longer possible.		
	f) Feedback was helpful for the analysis.		

General			
Proximal contacts	able to be passed through).		
	2. Proximal contacts are broken too broadly (over-extended) or proximal boxes are too wide		
	3. Proximal contacts are not sufficiently broken or boxes are designed too narrow (Wtip of WHO probe cannot be passed through)		
	a) No correction is necessary.		
	b) Correction attempt after feedback was successful.		
	c) Correction attempt after feedback was unsuccessful.		
	d) Feedback was helpful for the correction.		
	e) Correction is no longer possible.		
	f) Feedback was helpful for the analysis.		
Integrity of adjacent teeth	1. No iatrogenic damage to the adjacent tooth/ teeth		
	2. Minor iatrogenic damage to the adjacent tooth/ teeth		
	3. Significant iatrogenic damage to the adjacent tooth / teeth		
	a) No correction is necessary.		
	b) Correction attempt after feedback was successful.		
	c) Correction attempt after feedback was unsuccessful.		
	d) Feedback was helpful for the correction.		
	e) Correction is no longer possible.		
	f) Feedback was helpful for the analysis.		
Occlusal reduction	1. Adequate occlusal reduction (1.5mm)		
	2. Over reduction occlusal reduction (well over 1.5mm)		
	3. Insufficient occlusal reductionl (<1.5mm)		
	a) No correction is necessary.		
	b) Correction attempt after feedback was successful.		
	c) Correction attempt after feedback was unsuccessful.		
	d) Feedback was helpful for the correction.		
	e) Correction is no longer possible.		
	f) Feedback was helpful for the analysis.		
Feather edge	1. Adequate transition from the cavity to the tooth surface in a feather edge (0.5-1mm)		
	2. Feather edge insufficiently pronounced		
	3. Feather edge is too pronounced		
	a) No correction is necessary.		
	b) Correction attempt after feedback was successful.		
	c) Correction attempt after feedback was unsuccessful.		
	d) Feedback was helpful for the correction.		
	e) Correction is no longer possible.		
	f) Feedback was helpful for the analysis.		
