# Supplementary material for: Reduction of discrepancies between students and instructors in the assessment of practical tasks through structured evaluation sheets and peer feedback
Source: Sci Rep. 2024 Jan 17;14:1514. doi: 10.1038/s41598-024-51953-4 (PMC10794213; doi:10.1038/s41598-024-51953-4)
Supplement: Supplementary file 5 — Supplementary Information 5. [file 41598_2024_51953_MOESM5_ESM.pdf]

**Semester**	**Name of student:**	Stud.	peer
**Task**	**Partial crown - ceramic**		
**Tooth**	26		

**Cavity design**			
Cavity extention	1. Adequate extent of the cavity (2-3mm width and depth)		
	2. Over-extention of the cavity (>2-3mm width and depth)		
	3. Under-extention (correctable) of the cavity (<2-3mm width and depth)		
	a) No correction is necessary.		
	b) Correction attempt after feedback was successful.		
	c) Correction attempt after feedback was unsuccessful.		
	d) Feedback was helpful for the correction.		
	e) Correction is no longer possible.		
	f) Feedback was helpful for the analysis.		
Cavity walls	1. Adequate divergence (6-10°)		
	2. Inadequate divergence (<6°)		
	3. Over divergence (>6°)		
	a) No correction is necessary.		
	b) Correction attempt after feedback was successful.		
	c) Correction attempt after feedback was unsuccessful.		
	d) Feedback was helpful for the correction.		
	e) Correction is no longer possible.		
	f) Feedback was helpful for the analysis.		
Cavity floor	1. Flat cavity floor, corresponding to the load axis of the tooth		
	2. Slight roughness along the cavity floor		
	3. Significant roughness along the cavity floor		
	a) No correction is necessary.		
	b) Correction attempt after feedback was successful.		
	c) Correction attempt after feedback was unsuccessful.		
	d) Feedback was helpful for the correction.		
	e) Correction is no longer possible.		
	f) Feedback was helpful for the analysis.		
Proximal box depth	1. Adequate depth (2-3mm depth)		
	2. The design of the box is too deep (>2-3mm depth)		
	3. Insufficient depth of the box (<2-3mm depth)		
	a) No correction is necessary.		
	b) Correction attempt after feedback was successful.		
	c) Correction attempt after feedback was unsuccessful.		
	d) Feedback was helpful for the correction.		
	e) Correction is no longer possible.		
	f) Feedback was helpful for the analysis.		
Box walls	1. Adequate divergence including 'bicycle path' (6-10°)		
	2. Inadequate divergence (<6°)		
	3. Over divergence (>6°)		
	a) No correction is necessary.		
	b) Correction attempt after feedback was successful.		
	c) Correction attempt after feedback was unsuccessful.		
	d) Feedback was helpful for the correction.		
	e) Correction is no longer possible.		
	f) Feedback was helpful for the analysis.		

	1. Flat box floor, corresponding to the load axis of the tooth		
	2. Slight roughness along the box floor		
	3. Significant roughness along the box floor		

Box floor	a) No correction is necessary.		
	b) Correction attempt after feedback was successful.		
	c) Correction attempt after feedback was unsuccessful.		
	d) Feedback was helpful for the correction.		
	e) Correction is no longer possible.		
	f) Feedback was helpful for the analysis.		

**General**			
Proximal contact	able to be passed through).		
	2. Proximal contacts are broken too broadly (over-extended) or proximal box is too wide		
	3. Proximal contacts are not sufficiently broken (tip of WHO probe cannot be passed through/ under-extended) or proximal box is too narrow		
	a) No correction is necessary.		
	b) Correction attempt after feedback was successful.		
	c) Correction attempt after feedback was unsuccessful.		
	d) Feedback was helpful for the correction.		
	e) Correction is no longer possible.		
	f) Feedback was helpful for the analysis.		
Integrity of adjacent teeth	1. No iatrogenic damage to the adjacent tooth/ teeth		
	2. Minor iatrogenic damage to the adjacent tooth/ teeth		
	3. Significant iatrogenic damage to the adjacent tooth/ teeth		
	a) No correction is necessary.		
	b) Correction attempt after feedback was successful.		
	c) Correction attempt after feedback was unsuccessful.		
	d) Feedback was helpful for the correction.		
	e) Correction is no longer possible.		
	f) Feedback was helpful for the analysis.		
Occlusal reduction (including shortening of the cusps)	1. Adequate reduction of all occlusal surfaces (at least 2mm)		
	2. Over reduction of the occlusal surfaces (>4mm)		
	3. Insufficient reduction of the occlusal surfaces (<2mm)		
	a) No correction is necessary.		
	b) Correction attempt after feedback was successful.		
	c) Correction attempt after feedback was unsuccessful.		
	d) Feedback was helpful for the correction.		
	e) Correction is no longer possible.		
	f) Feedback was helpful for the analysis.		
Right-angled transition to the tooth surface	1. Adequate transition from the cavity to the tooth surface (90°)		
	2. Feather edge (in places), contraindicated for the material		
	a) No correction is necessary.		
	b) Correction attempt after feedback was successful.		
	c) Correction attempt after feedback was unsuccessful.		
	d) Feedback was helpful for the correction.		
	e) Correction is no longer possible.		
	f) Feedback was helpful for the analysis.		
