# Supplementary material for: Reduction of discrepancies between students and instructors in the assessment of practical tasks through structured evaluation sheets and peer feedback
Source: Sci Rep. 2024 Jan 17;14:1514. doi: 10.1038/s41598-024-51953-4 (PMC10794213; doi:10.1038/s41598-024-51953-4)
Supplement: Supplementary file 6 — Supplementary Information 6. [file 41598_2024_51953_MOESM6_ESM.pdf]

OA Dr Andreas Vahlenkamp

Alfred-Herrhausen-Str. 50
D-58448 Witten
Telephone 02302/926-0
Fax 02302/926-661

Integrated preclinical course
Department of Dentistry, Oral and Maxillofacial Medicine
Faculty of Health

University of Witten/Herdecke, 58448 Witten

# Evaluation sheet in the integrated preclinical course preparatory program (traditional Sheet)

## Cavity Preparations and fillings

| | mod | |
|---|---|---|
| Tooth | 36 | |
| | E | A |
| Compliance with the preparation characteristics | | |
| Integrity of adjacent teeth | | |
| **Evaluation** | | |

| | mvpi | |
|---|---|---|
| Tooth | 21 | |
| | E | A |
| Compliance with the preparation characteristics | | |
| Integrity of adjacent teeth | | |
| **Evaluation** | | |

## Partial crown preparation 46 und 26

| | 46 | | 26 | |
|---|---|---|---|---|
| **Evaluation criteria** | E | A | | |
| Anatomically correct preparation | | | | |
| Removal of contact points | | | | |
| Maintaining the integrity of the adjacent teeth | | | | |
| Preparation characteristics followed | | | | |
| Surface design/finish | | | | |
